# Geriatric Nutritional Risk Index Predicts Prognosis of Elderly Gastric Cancer Patients After Non-Curative Endoscopic Treatment

**DOI:** 10.24546/0100504447

**Published:** 2026-06-02

**Authors:** HITOSHI HARADA, SHINGO KANAJI, YUTAKA SUGITA, TARO IKEDA, YASUFUMI KOTERAZAWA, TOMOAKI AOKI, YASUNORI OTOWA, NAOKI URAKAWA, HIRONOBU GOTO, HIROSHI HASEGAWA, TOSHITATSU TAKAO, MASASHI YAMAMOTO, KIMIHIRO YAMASHITA, TAKERU MATSUDA, YUZO KODAMA, YOSHIHIRO KAKEJI

**Affiliations:** 1 Division of Gastrointestinal Surgery, Department of Surgery, Kobe University Graduate School of Medicine, Kobe, Japan; 2 Division of Gastroenterology, Department of Internal Medicine, Kobe University Graduate School of Medicine, Kobe, Japan; 3 Ninokiri Yamamoto Clinic, Osaka, Japan; 4 Division of Analytical Biomedical Sciences, Department of Biophysics, Kobe University Graduate School of Health Sciences, Kobe, Japan

**Keywords:** Gastric cancer, Endoscopic submucosal dissection, Gastrectomy, Nutritional risk index, Elderly

## Abstract

Additional gastrectomy is usually performed after endoscopic submucosal dissection (ESD) for early gastric cancer diagnosed as “non-curative” according to the Japanese guidelines. However, the prognostic benefit of additional gastrectomy for the elderly with non-curative ESD is still debatable. The Geriatric Nutritional Risk Index (GNRI) is known as a prognostic value in elderly patients. This study aimed to clarify the advantages of GNRI as a prognostic indicator for elderly gastric cancer patients with non-curative ESD. A total of 120 elderly patients who underwent ESD for gastric cancer and diagnosed as non-curative resection according to the Japanese guidelines were retrospectively selected from the database of the Kobe University Hospital between January 2008 and March 2020. Their general condition, risk of lymph node metastasis, and nutritional status were evaluated retrospectively, and prognostic analysis was performed. After non-curative ESD, 48 patients underwent additional gastrectomy with lymph node dissection, and 72 patients were followed up with no additional treatment. In the follow-up group, patients with a high risk of lymph node metastasis had a significantly poorer prognosis. However, in the prognostic analysis of 120 cases, additional gastrectomy or follow-up were not independent prognostic factors. In the analysis of the additional gastrectomy group only, major nutrition-related risk of postoperative GNRI was an independent prognostic factor (95% CI: 0.01–0.25, P < 0.001). In elderly, the GNRI may be helpful as a prognostic indicator. Especially for elderly patients with poor nutritional status, additional gastrectomy might be considered only in cases of high risk of recurrence after non-curative ESD.

## INTRODUCTION

While radical gastrectomy with lymph node dissection provides curative benefits to gastric cancer patients, postoperative weight loss and malnutrition are considered disadvantages [1, 2]. Additional gastrectomy is usually performed for early gastric cancer after endoscopic submucosal dissection (ESD), which is pathologically presumed to be at high risk of lymphatic metastasis (diagnosed as “non-curative” according to Japanese guidelines) [3] according to Japanese gastric cancer treatment guidelines [4]. However, elderly patients (especially those over 75 years old) may be at an elevated risk of potential morbidity and mortality due to multiple prognostic comorbidities [5, 6]. The negative prognostic consequences of additional gastrectomy, such as surgical invasiveness and a significant decrease in postoperative performance, cannot be ignored, especially for elderly patients. A controversy exists on whether a standardized additional gastrectomy for elderly gastric cancer patients who are diagnosed with non-curative resection after ESD has a prognostic advantage. Therefore, it is essential to predict the prognostic benefits of additional gastrectomy and the prognostic disadvantages of surgical invasiveness in elderly patients.

The Geriatric Nutritional Risk Index (GNRI), which indicates nutritional status and is derived from serum albumin levels and body weight, is a prognostic value for the elderly [7, 8]. GNRI is a simple and useful value for predicting all-cause mortality, especially in elderly patients with heart failure [9–11]. GNRI may sensitively reflect preoperative and post-gastrectomy nutritional status in elderly patients with gastric cancer because its value is calculated from albumin and body weight. Therefore, it might also be useful as a prognostic value in evaluating the nutritional disadvantages of gastrectomy in elderly patients. However, the actual advantage of the GNRI as a prognostic indicator in elderly patients diagnosed with non-curative resection after ESD for early gastric cancer remains unknown. The aim of this study was to clarify the advantage of the GNRI as a prognostic indicator to predict the risk of additional gastrectomy for elderly early gastric cancer patients diagnosed with non-curative resection after ESD.

## MATERIALS AND METHODS

Elderly patients who underwent non-curative ESD (defined as “eCuraC” in Japanese gastric cancer treatment guidelines [4]) for gastric cancer were selected from the database of the Kobe University Hospital between January 2008 and March 2020 retrospectively. The patient eligibility criteria were as follows: age 75 years or older, pathologically diagnosed adenocarcinoma, eCuraC diagnosis after ESD, and no other coexisting malignant disease at the time of ESD. This study enrolled a total of 120 patients, of which 48 underwent additional gastrectomy with lymph node dissection (additional gastrectomy group), and 72 were followed up but received no additional treatment after ESD (follow-up group), based on clinician decisions.

### Patient characteristics and clinical findings

Patient characteristics included age, sex, tumor size, histology, presence of venous or lymphatic invasion, and positive vertical margin (Table I). The patients’ general condition was evaluated using the American Society of Anesthesiologists Physical Status (ASA PS) and Charlson Comorbidity Index (CCI) [12].

Hatta et al. recommended additional gastrectomy for patients who are diagnosed in the high-risk category by scoring with the eCura system, based on the significant correlation between recurrence and cancer-related death [13]. Scoring using the eCura system was used to evaluate the risk of lymph node metastasis. The patient population was stratified by scoring using the eCura system, and a prognostic analysis was performed.

The GNRI at the time of ESD was also calculated from blood examination data as an assessment of nutritional status. The GNRI was calculated using serum albumin level as follows: [1.489 × serum albumin level (g/L)] + [41.7 × (present/ideal body weight (kg))] [7, 8]. Ideal body weight of male and female are defined as: height (cm) − 100 − [(height (cm) − 150)/4] (kg) for male and height (cm) − 100 − [(height (cm) − 150)/2.5] (kg) for female. Four stages of nutrition-related risk were defined according to the previous study: Major risk: GNRI <82; moderate risk: GNRI 82 to <92; low risk: GNRI 92 to ≤98; and no risk: GNRI >98 [7].

For patients who underwent additional gastrectomy, types of surgical procedure, pathological findings (residual tumor and lymph node metastasis), postoperative complications, rate of weight loss at 6 months postoperatively, and GNRI 6 months after gastrectomy (postoperative GNRI) were additionally examined.

The eighth UICC/AJCC TNM classification was used for clinicopathological evaluation [14]. Postoperative complications were based on the Clavien-Dindo classification [15].

### Follow-ups and overall survivals

All patients were usually followed up using annual esophagogastroduodenoscopy and computed tomography. During the observation period of each patient, data on five-year overall survival (OS) were collected. The OS time was defined as the time from ESD to death for any reason or interruption of follow-up. The median follow-up period was 4.23 years (IQR 3.01–6.03) for all patients, 4.20 years (IQR 2.95–6.03) in the additional gastrectomy group, and 4.27 years (IQR 3.03–6.09) in the follow-up group. Two patients in the follow-up group were lost to follow-up.

### Statistical analyses

The χ^2^ test or Fisher’s exact test was applied to evaluate each dichotomous variable, and the Wilcoxon rank-sum test was applied for continuous variables. OS was estimated using the Kaplan-Meier method. *P* values < 0.05 were considered statistically significant. Data were analyzed using JMP software (v. 13; SAS Institute Inc., Cary, NC, USA).

## RESULTS

The enrolled patients aged >75 years who underwent non-curative ESD comprised 72 patients with no additional treatment and 48 with radical gastrectomy. Patient characteristics showed that those with older age, ASA PS ≥ 3, CCI ≥ 5, or worse nutritional status were significantly more likely to have no additional gastrectomy. In contrast, significantly more patients underwent additional gastrectomy for submucosal or deeper invasion (≥pT1b) or lymphatic invasion in the pathological findings after ESD. Correlatively, additional gastrectomy was performed in significantly more cases with a high risk of lymph node metastasis according to the eCura system (≥5 points). The follow-up group had a significantly higher proportion of patients classified as having major nutrition-related risk by the GNRI (<82) at the time of ESD (Table I). Both the follow-up and additional gastrectomy groups had three deaths each, but the incidence rates were not significantly different. On the other hand, deaths due to causes other than gastric cancer, occurred in 8 cases in the additional gastrectomy group and 30 cases in the follow-up group (Table II).

**Fig. 1:**
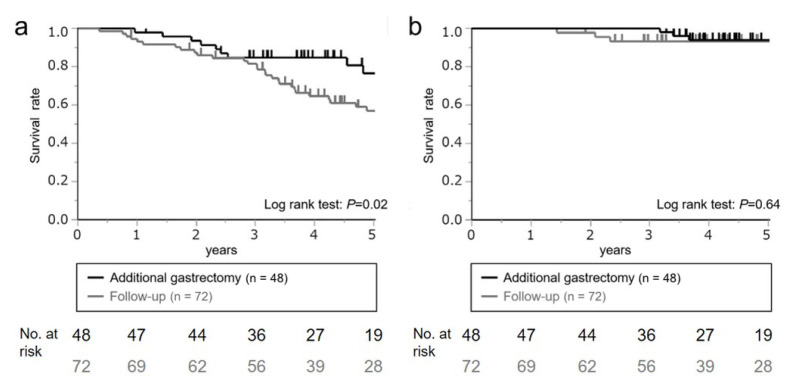
(a) Comparison of overall survival between patients with no additional treatment and those with additional surgery after ESD (b) Comparison of cancer-specific survival between patients with no additional treatment and those with additional surgery after ESD

**Fig. 2:**
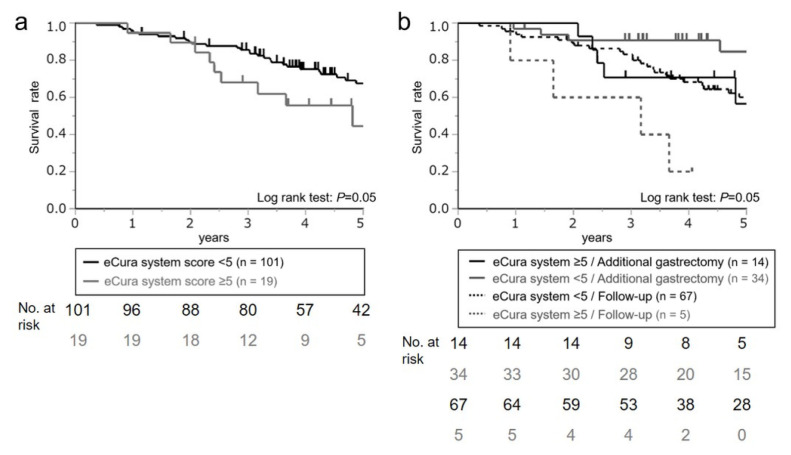
(a) Comparison of overall survival between two groups of patients categorized according to the risk categories of the eCura system (b) Comparison of overall survival between four groups of patients categorized according to the risk categories of the eCura system and treatment

Patients who underwent additional gastrectomy had a better prognosis than those were followed up with no additional treatment (Fig. 1A). However, when cancer-specific survival rates between patients in the follow-up and additional gastrectomy groups were compared, no significant difference was observed (Fig. 1B). Patients at high risk for the eCura system had a poor prognosis (Fig. 2A). Furthermore, comparing survival curves based on a combination of eCura system score and whether surgery was performed revealed that patients with high eCura system risk and no additional treatment had worse prognosis (Fig. 2B).

**Fig. 3:**
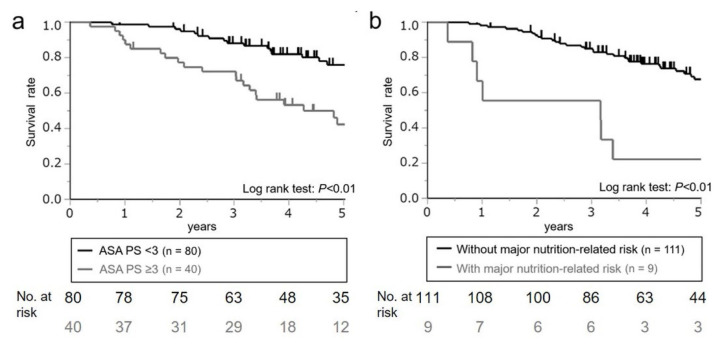
(a) Comparison of overall survival between two groups categorized general condition risk according to the ASA PS (b) Comparison of overall survival between two groups categorized according to the nutritional risk categories of the preoperative GNRI

In terms of ASA PS and GNRI, patients with a poor general condition (ASA PS ≥ 3) and those with severe malnutrition (GNRI < 82) both had a significantly poorer prognosis (Fig. 3A and 3B). In the univariate analysis of overall survival, the prognosis was significantly worse in cases with no additional gastrectomy, ASA PS ≥ 3, CCI ≥ 5, positive vertical margin, and GNRI < 82. No significant difference in prognosis was found between those who were categorized as high risk for lymph node metastasis by the eCura system scoring and those who were categorized as intermediate risk or lower. In multivariate analysis, ASA PS ≥ 3 and GNRI < 82 were independent poor prognostic factors (95% CI: 1.09–4.35, *P* = 0.02; 95% CI: 0.09–6.25, *P* = 0.03, respectively) (Table III).

### Analysis of cases with additional gastrectomy

**Fig. 4:**
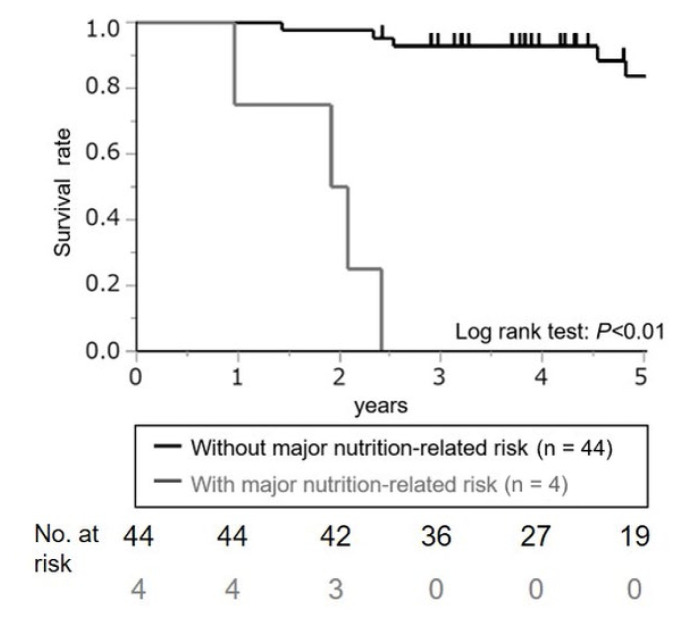
Comparison of overall survival between two groups categorized according to the nutritional risk categories of the postoperative GNRI in patients with additional gastrectomy

To determine the disadvantages of additional gastrectomy, such as surgical invasiveness and decreased performance, we analyzed 48 patients in the additional gastrectomy group. Of these, three patients had complications of Clavien-Dindo classification 3 or higher, but no operation-related deaths occurred. Pathological findings after gastrectomy revealed post-ESD residual tumor in eight patients and regional lymph node metastasis in three patients (Table IV). Patients who underwent additional gastrectomy had weight loss rates ranging from 1.3–31.7% (median: 11.9%). No cases with preoperative GNRI showed major nutritional risk, but four cases turned into major nutrition-related risk 6 months postoperatively (postoperative GNRI). All four patients with major nutrition-related risk in postoperative GNRI had a significantly poor prognosis and fatal outcome (Fig. 4). In univariate analysis, preoperative ASA PS ≥ 3, major nutrition-related risk of postoperative GNRI (<82), and pathological regional lymph node metastasis positivity (pN+) were significantly poor prognostic predictors. In the multivariate analysis, the major nutrition-related risk of postoperative GNRI was an independent prognostic factor (Table V).

## DISCUSSION

Due to the prevalence of multiple prognostic comorbidities, elderly patients are generally at an elevated risk for potential morbidity and mortality [5, 6]. Suzuki et al. showed that postoperative complications, such as severe pneumonia, are responsible for worsening the long-term survival of elderly patients with gastric cancer [16]. However, research shows that elderly patients with various comorbidities can safely undergo minimally invasive surgery [17–20]. Therefore, the short-term invasiveness of gastrectomy is not considered to be significantly related to prognosis.

In this study, the survival curves showed that the additional gastrectomy group had a better prognosis than the follow-up group. However, the patient characteristics showed that a higher rate of patients in the follow-up group were older and had worse general condition and nutritional status than those in the additional gastrectomy group. In the cancer-specific survival rate, prognosis in the additional gastrectomy and follow-up groups did not differ significantly. This study was a retrospective evaluation, and the decision to perform an additional gastrectomy or follow-up after ESD was based on the clinician’s evaluation of the individual patient’s condition. The bias due to each clinician’s decision might be associated with the difference in overall survival rates between the two groups.

Compared to patients with eCura system scores of less than 5, patients with higher eCura system scores tended to have a worse prognosis, although the difference was not statistically significant. Two patients of the three cancer-related deaths in the follow-up group were diagnosed as having a high risk of lymph node metastasis with eCura system scores of 5 or higher. In addition, a comparison between survival curves based on a combination of the eCura system score and additional gastrectomy showed that patients with high eCura system scores and no additional gastrectomy had a significantly poor prognosis. This result suggests that additional gastrectomy might be performed even in cases of elderly patients who are at severe risk of non-curative factors. Therefore, scoring using the eCura system might be appropriate as an indicator for the validity of additional gastrectomy for non-curative resection cases after ESD in elderly patients with gastric cancer.

However, in the prognostic analysis of 120 cases, additional gastrectomy or follow-up was not an independent prognostic factor, whereas ASA PS and GNRI were independent prognostic factors. Therefore, follow-up without additional treatment might be an acceptable option for elderly patients with gastric cancer who have a low or intermediate risk of non-curative factors after ESD. Hatta et al. reported a cancer-specific survival rate at 5 years as 90.1% in high-risk cases by eCura system scoring (≥5 points) with no additional gastrectomy [13]. In contrast, in this study, the overall survival rates at 5 years for patients who have a poor ASA PS and major nutrition-related risk by GNRI were 40% and 20%, respectively. These results might indicate that a severe decline in the general condition and malnutrition are more significant prognostic risks in elderly gastric cancer patients with non-curative resection after ESD. Hence, ASA PS and GNRI may be potentially useful prognostic predictors.

Additionally, the GNRI can gradually represent changes in nutritional status following gastrectomy because its value is derived from body weight and serum albumin levels. Malnutrition resulting from decreased nutritional intake is a typical sequela of gastrectomy, and postoperative malnutrition has been shown to be a predictor of poor prognosis [21]. In this study, the additional gastrectomy group did not include patients with a major nutrition-related risk of GNRI preoperatively. Nevertheless, despite the absence of nutritional risk on preoperative GNRI, four cases showed remarkable weight loss and hypoalbuminemia 6 months postoperatively and a rapid decline in GNRI. All four patients with major nutrition-related risk with postoperative GNRI had a poor prognosis and a fatal outcome. Therefore, the GNRI might be beneficial for preoperative risk assessment as well as a follow-up indicator of prognostic risk after gastrectomy, indicating the decline in postoperative nutritional status.

This study had several limitations. First, this was a single-center, retrospective study with a small sample size. Second, in elderly gastric cancer patients diagnosed with eCuraC after ESD, the decision to follow up with no additional treatment or perform an additional gastrectomy is largely dependent on the attending physician’s assessment, including the patient’s decision in a particular case. In this study, patients were not randomly assigned, and a considerable selection bias was noted. Third, regarding cancer-related deaths from additional gastrectomy, two out of the three recurrent cases had advanced gastric cancer, and one was a pathologically special type (mixed adenoneuroendocrine carcinoma). This study included cases with potentially poor prognosis, even with radical gastrectomy.

In conclusion, the GNRI may serve as a useful prognostic indicator reflecting perioperative nutritional status for elderly patients. Additional gastrectomy after non-curative ESD might be considered only for patients at high risk of recurrence. In particular, it may be better avoided in elderly patients with poor general condition, and nutritional assessment may help inform individualized decision-making.

## Figures and Tables

**Table I:** Clinicopathological characteristics of patients after non-curative endoscopic submucosal dissection for gastric cancer

Variable		Additional gastrectomy (n = 48)	Follow-up (n = 72)	*P*-value
Age	Median (range)	79 (75–87)	82 (75–92)	<0.001
Gender	Male/Female	40/8	53/19	0.20
ASA PS	<3/≥3	38/10	42/30	0.01
CCI	<5/≥5	32/16	30/42	0.006
Tumor size (mm)	Median (range)	24.5 (9–85)	28 (4–132)	0.11
Histopathological type	differ/undiffer/other	39/8/1	66/6/0	0.14
Tumor depth	<T1b2/≥T1b2	12/36	36/36	0.005
Lymphatic invasion (Ly)	−/+	17/31	56/16	<0.001
Venous invasion (V)	−/+	38/10	66/6	0.05
Vertical margin (VM)	−/+	38/10	60/12	0.56
eCura system score	0–4/5–7	34/14	67/5	0.001
GNRI^a^	No risk/Low or Moderate risk/Major risk	32/16/0	31/32/9	0.001

a In the additional gastrectomy group, refer to preoperative score.

ASA PS, American society of anesthesiologists physical status; CCI, Charlson Comorbidity Index; GNRI, Geriatric Nutritional Risk Index.

**Table II:** Univariate and multivariate predictors of overall survival of patients after non-curative endoscopic submucosal dissection (eCuraC)

Variable	Additional gastrectomy	Follow-up
Cancer death (gastric cancer)	3	3
Death of non-gastric cancer causes	8	30
Other malignancies	0	5
Heart failure	0	3
Hepatic failure	0	3
Pneumoniae	0	5
Acute kidney injury	0	1
Aortic dissection	0	1
Cerebral infarction	0	1
Other disease	7	11

**Table III:** Univariate and multivariate predictors of overall survival of patients after non-curative endoscopic submucosal dissection (eCuraC)

Variable		n = 120	Univariate			Multivariate		

			HR	95% CI	*P*-value	HR	95% CI	*P*-value
Treatment after ESD	Follow up	72	2.17	1.10–4.35	0.02	1.79	0.78–4.17	0.17
	Gastrectomy	48						
Age	≥80	70	1.64	0.89–3.00	0.11			
	75–79	50						
Gender	Male	93	1.39	0.68–2.86	0.36			
	Female	27						
ASA PS	≥3	40	3.57	1.96–6.67	<0.001	2.17	1.09–4.35	0.02
	<3	80						
CCI	≥5	58	3.57	1.85–6.67	<0.001	1.89	0.87–4.17	0.11
	<5	62						
Tumor size (mm)	>30	50	1.33	0.74–2.38	0.34			
	≤30	70						
Histopathological type	Undiffer/Other	15	4.15	0.57–30.2	0.16			
	Differ	105						
Tumor depth	≥T1b2	72	1.15	0.64–2.08	0.64			
	<T1b2	48						
Lymphatic invasion (Ly)	+	47	1.32	0.73–2.38	0.37			
	−	73						
Venous invasion (V)	+	16	1.07	0.42–2.72	0.87			
	−	104						
Vertical margin (VM)	+	22	2.00	1.04–3.85	0.03	1.69	0.83–3.57	0.15
	−	98						
eCura system score	≥5	19	2.00	0.97–4.00	0.06			
	0–4	101						
GNRI^a^	Major risk (<82)	9	4.00	1.75–9.09	<0.001	2.56	1.09–6.25	0.03
	No to Moderate risk (≥82)	111						

a In the additional gastrectomy group, refer to preoperative score.

ASA PS, American society of anesthesiologists physical status; CCI, Charlson Comorbidity Index; GNRI, Geriatric Nutritional Risk Index.

**Table IV:** Clinical characteristics of patients after non-curative ESD for gastric cancer who underwent additional gastrectomy

Variable		n = 48
Approach	Open	3
	Laparo	45
Procedure	DG	38
	PG	8
	TG	2
% Weight Loss (Median (Range))		11.9 (1.3–31.7)
	<20	34
	≥20	14
Postoperative complications^a^	≤II	45
	≥III	3
GNRI		
Preoperative	No to Moderate risk (≥82)	48
	Major risk (<82)	0
Postoperative	No to Moderate risk (≥82)	44
	Major risk (<82)	4
Residual tumor	−	40
	+	8
Lymph node metastasis	pN0	45
	pN+	3
Cancer death	−	45
	+	3

a According to Clavien-Dindo classification.

GNRI, Geriatric Nutritional Risk Index.

**Table V:** Univariate and multivariate predictors of overall survival of patients who received additional gastrectomy after noncurative endoscopic submucosal dissection (eCuraC)

Variable		n = 48	Univariate			Multivariate		

			HR	95% CI	*P*-value	HR	95% CI	*P*-value
ASA PS	≥3	10	4.00	1.04–16.67	0.04	1.23	0.16–9.19	0.83
	<3	38						
CCI	≥5	16	2.04	0.55–7.69	0.29			
	<5	32						
Procedure	TG	2	3.85	0.45–33.33	0.22			
	non-TG	46						
% Weight Loss	≥20	14	2.56	0.76–9.09	0.13			
	<20	34						
Postoperative GNRI^a^	<82	4	42.3	7.34–243.8	<0.001	41.7	3.95–441.2	<0.001
	≥82^b^	44						
Residual tumor-	+	8	1.52	0.38–6.25	0.56			
	40							
Lymph node metastasis	pN0	45	6.67	1.25–33.3	0.02	2.38	0.36–16.67	0.37
	pN+	3						
Postoperative complications^c^	≥III	3	5.56	0.66–50.00	0.11			
	≤II	45						

a Geriatric Nutritional Risk Index at 6 months postoperatively.

b Major nutrition-related risk in Geriatric Nutritional Risk Index.

c According to Clavien-Dindo classification.

TG, Total gastrectomy; ASA PS, American society of anesthesiologists physical status; CCI, Charlson Comorbidity Index; GNRI, Geriatric Nutritional Risk Index.
